# Distractor Inhibition Predicts Individual Differences in Recovery from the Attentional Blink

**DOI:** 10.1371/journal.pone.0064681

**Published:** 2013-05-21

**Authors:** Heleen A. Slagter, Katerina Georgopoulou

**Affiliations:** 1 Department of Psychology, University of Amsterdam, Amsterdam, The Netherlands; 2 Cognitive Science Center Amsterdam, University of Amsterdam, Amsterdam, The Netherlands; University of Groningen, The Netherlands

## Abstract

**Background:**

The attentional blink (AB) refers to an impairment in detecting the second of two target stimuli presented in close succession in a rapid stream of distractors. Recent studies indicate that the AB results, in part, from distractor suppression mechanisms, that may be mediated by striatal dopamine. Yet, it is currently unclear how distractor suppression ability may contribute to the AB. Here, we examined whether distractor suppression ability is predictive of an individual's AB depth and/or recovery. In addition, we investigated the relationship between individual spontaneous eye blink rate (sEBR), a marker of striatal dopaminergic functioning, and AB performance.

**Methodology/Principal findings:**

Subjects were presented with rapid streams of letters containing white distractors, a red T1 and a green T2. T2 was presented either at Lag2, Lag4 or Lag10, and preceded by a distractor that shared the same identity as T2 (T2 primed) or not (T2 not primed). Replicating previous work [1], we found that slow AB recovery (poor T2 performance in Lag4 vs. Lag10) was associated with a failure to inhibit distractors, as indexed by greater positive priming. However, no relationship was observed between a subject's ability to suppress distractors and AB depth (Lag10 vs. Lag2). Moreover, no relationship between sEBR and AB performance was observed.

**Results/Significance:**

These results indicate that a failure to inhibit distracting information impairs AB recovery, possibly by interfering with target encoding in working memory - but does not affect AB magnitude. The absence of a relationship between individual sEBR and AB performance may be explained by task specifics.

## Introduction

In an ever-changing world, our senses are continuously bombarded with more information than our brain can process up to the level of awareness. Hence, the ability to rapidly select goal-relevant information when it occurs, while simultaneously inhibiting irrelevant or distracting information is central to goal-directed behavior. The challenge our brain faces when presented with an overwhelming amount of information to analyze is well captured by one of the most studied attentional phenomena in the literature: the so-called attentional blink deficit [2]. This deficit occurs when people have to detect two target stimuli (T1 and T2) presented in close temporal succession in a rapid (∼10 Hz) stream of distracter events. Specifically, people often fail to identify T2 when it follows T1 within 200–500 ms. Many models have been proposed to explain this deficit in target processing (for recent reviews, see [3], [4]), with some attributing the AB to T1-induced depletion of limited processing resources critical for consolidating a stimulus in working memory (capacity-based models; e.g., [5], [6]), and others explaining the AB in terms of dysfunctional gating of information *to* working memory rather than a capacity limitation *of* working memory (selection-based models; e.g., [7], [8]). While it is clear that the AB is related to having to encode a first target, the important contribution of the post-T1 distractor to the emergence of an AB is also widely recognized. In some accounts, a failure to suppress distractor stimuli impairs target selection. For example, it has been proposed that interference from distractor stimuli may delay T1 consolidation processes in working memory, decreasing the chances that this processing stage - which can only process one item at a time - becomes available in time for T2 consolidation [5]. It has alternatively been proposed that the AB - rather than a failure of distractor inhibition – reflects an inhibitory response meant to suppress the post-T1 distractor, but which accidentally suppresses the subsequently presented T2 [8].

A converging body of research supports an important role for distractor suppression mechanisms in the AB (e.g., [9]–[17]). For example, AB magnitude is predicted by the ability to keep irrelevant information out of working memory [12]. Yet, in this and many other studies, distractor suppression ability was measured indirectly, e.g., during performance of a different task. In a recent study by Dux and Marois [1], subjects' ability to suppress distractors was assessed during the rapid presentation conditions of the AB task itself. Specifically, subjects' ability to suppress distractors was assessed by determining the extent to which their T2 performance was primed by a preceding distractor that shared the same identity as T2. It was found that an individual subjects' magnitude of T2 priming from this distractor was positively correlated with their AB magnitude. That is, subjects with attenuated ABs showed negative priming (worse T2 performance after a priming vs. non-priming distractor), whereas those with large ABs displayed positive priming (better T2 performance after a priming distractor). As positive priming is indicative of reduced distracter inhibition, it was concluded that the AB results, at least in part, from a failure of distractor inhibition.

It should be noted, however, that in the Dux and Marois [1] study, T2 was presented either at Lag4 or at Lag10, and AB magnitude was defined as the difference in T2 performance in Lag10 vs. Lag4 trials. Yet, the AB is typically largest at Lag2 or Lag3, and individuals generally display significant recovery of T2 performance at Lag4 [5]. It is hence possible that a failure to inhibit distracting information primarily affected *recovery* from the AB rather than AB *magnitude*. The first aim of the current study was therefore to examine the extent to which subjects' ability to suppress distractors as measured during AB task performance predicts individual differences in AB magnitude and/or AB recovery.

The second aim of this study was to further our understanding of the relationship between striatal dopamine and the AB. Striatal dopamine is thought to play a key role in regulating the contents of working memory by gating relevant information to prefrontal working memory and preventing gating of irrelevant information (e.g., [18]–[20]). It is notable in this respect that several studies have recently related indices of striatal dopamine to AB task performance [21]–[23] (but see [24]), suggesting that mechanisms that control access to working memory represent an important mechanism underlying the AB. For example, a recent positron emission tomography (PET) study by Slagter et al. [21] showed that individual differences in striatal dopamine D2 receptor binding predicted individual AB size. Of further importance, Colzato et al. [22] found that those individuals who blinked more often spontaneously (with their eyes) generally displayed a smaller AB. Spontaneous eye blink rate (sEBR) has been consistently related to striatal dopamine levels in both animals and humans (e.g., [25]–[28]), and the observed relationship between sEBR and AB size thus further indicates a role for striatal dopamine in the AB. Here, we aimed to replicate and extend this latter finding, and examined the relationship between sEBR and both AB magnitude and AB recovery.

## Materials and Methods

### Ethics Statement

The ethical committee of the Department of Psychology of the University of Amsterdam approved the experiment and written consent was obtained from the subjects after the nature and possible consequences of the study were explained to them.

### Participants, Procedure, and Conditions

45 subjects (22 female; mean age 22,6 years) participated in the study. They had normal or corrected-to-normal sight, no history of neurological or psychiatric disorders, and were not color blind. Subjects participated for research credit or money (7 euros per hour).

After subjects provided written consent, their spontaneous eye blinks were recorded with two vertical Ag–AgCl electrodes above and below the left eye, for 4-min eyes-open segments under resting conditions (cf. [22], [29], [30]). A ground electrode was placed on the forehead. Given that spontaneous EBR is stable during daytime, but increases in the evening [31], data were never collected after 5 p.m. In addition, we asked participants to avoid alcohol and nicotine consumption and to sleep sufficiently the day before the recording. During recordings, participants did not wear contact lenses, were alone in the room, and sat upright and silent. They were asked to look straight ahead at a white wall about 1.5 meters in front of them, and were not instructed in any manner about blinking.

**Figure 1:**
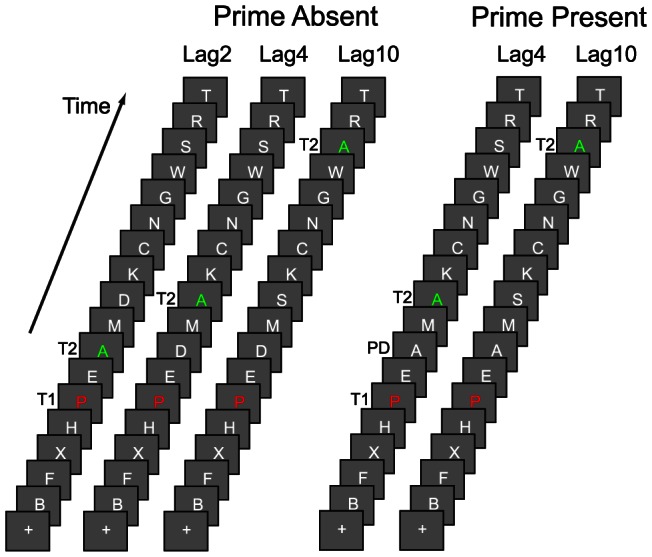
Attentional blink task. Subjects viewed RSVP streams of letters. Target 1 (T1) was colored red, Target 2 (T2) green, and the distractors white. T2 could appear at Lag 2, 4 or 10. In the prime present trials, a distractor (priming distractor, PD) with the same identity as T2 appeared at Lag2. All stimuli had different identities in the prime absent trials. Subjects were required to report T1 and T2 at the end of each stream.

After the sEBR recordings, subjects were seated approximately 90 cm from a computer screen in a comfortable chair. The 23-inch LCD high-performance gaming monitor was driven by a standard personal computer running the microsoft operating system XP and refreshed at 120 Hz with a resolution of 1920×1080 pixels in 16-bit color. They then performed an attentional blink task, which was modeled after the AB task used by Dux and Marois [1] (Figure 1). Each AB trial consisted of a rapid serial visual presentation of a sequence of uppercase letters drawn from the alphabet excluding I, L, O, Q, U, and V. T1 was red, T2 green, distractors white, and the background grey. T1 appeared at serial position 5, and T2 at Lag2, Lag4, and Lag10. A fixation square presented for 480 ms preceded all trials, while each stimulus appeared for 92 ms, with 17 of these stimuli presented in each trial. For the “prime absent” trials, all stimuli differed, while in the “prime present” trials the second distractor after T1 (at Lag2) had the same identity as T2 (priming distractor). This distractor appeared at the time of maximal blink (Lag2 [5]), so it was unlikely to be consciously perceived. Necessarily, priming distractors could only occur in trials in which T2 was presented at Lag4 or Lag10 (cf. [1]). Subjects typed the target identities when visually prompted at the conclusion of each stream. They performed 20 practice trials, and two blocks of 100 test trials each, with the five trial types randomly intermixed and equally probable. The task was programmed in Presentation. After the AB task, subjects performed a reinforcement learning task (data not reported here).

### Analysis

AB magnitude was defined as T2 accuracy given T1 correct (T2/T1) in Lag10 vs. Lag2 prime absent trials. AB recovery was defined as T2 accuracy given T1 correct (T2/T1) in Lag10 vs. Lag4 prime absent trials. T2/T1 accuracy was assessed in prime absent trials to get a measure of the AB that was independent of the prime (cf. [1]). Distractor priming magnitude was only assessed at Lag4, not Lag10, because of the short duration of RSVP priming ([32]; cf. [1]) and calculated as T2/T1 in prime present trials – T2/T1 in prime absent trials. Indeed, a paired t-test revealed no significant priming effect for Lag10 trials (t(39) = 1,1, p = .25)

Each individual's sEBR was computed according to automatic and manual procedures using Matlab (cf. [30]). First, a voltage threshold was determined that appeared to capture most blinks, and little artifacts (e.g., muscle-related artifacts) in the data. Then, 20-sec epochs were visually inspected for detection accuracy, i.e., the presence/absence of blinks. This resulted for each subject in a value reflecting their average spontaneous blink rate per minute (or sEBR).

Relationships between individual differences in AB performance, distractor suppression ability, and/or sEBR were examined using Spearman's rank correlation tests. We preferred Spearman's over Pearson's coefficient because of scale distortions in accuracy data, as well as the use of difference scores, resulting in data that are not normally distributed. Rank correlations are less sensitive to outliers and violations of the assumption of normally distributed data.

## Results and Discussion

Three individuals had to be excluded from analyses due to incorrect display refresh rate settings during AB task performance, and hence, incorrect stimulus presentation rates. Two additional individuals exhibited extremely low T1 accuracy rates (12% and 16%; more than 3 standard deviations from the mean), suggesting an inability or unwillingness to do the task, and were also excluded from analyses. In the remaining 40 subjects, T1 accuracy was on average 82%. More specifically, in prime absent trials, T1 accuracy was 79, 81 and 83% in, respectively, Lag2, Lag4, and Lag10 trials. In prime present trials, T1 accuracy was 82 and 83% in respectively, Lag4 and Lag10 trials.

**Figure 2:**
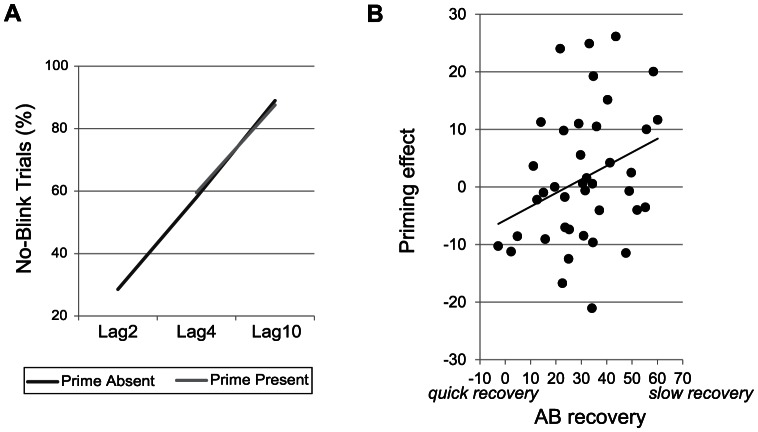
Behavioral results. A: Average AB performance data. T2/T1 accuracy data are shown for each condition (prime present/absent) and T1-T2 interval (Lag) separately. As can be seen, in both conditions, a substantial AB was observed. B: The observed relationships between distractor suppression ability and AB recovery. For illustrative purposes, the raw (rather than ranked) data are shown.

Figure 2A shows T2/T1 accuracy for each lag and condition (prime present/absent) separately. In both prime absent and prime present trials, a large AB was observed (main effect of Lag (2/4/10) in prime absent trials: F(1,39) = 260.6; p<.001; main effect of Lag (4/10) in prime present trials: F(1,39) = 114.0; p<.001). At the group level, priming did not affect AB performance, as indexed by a non-significant interaction between Condition and Lag (Lag4/Lag10) (F(1,39) = 1.8; p = .19). Yet, as in previous reports [1], there were large individual differences in both the size and direction of the priming effect, with some individuals benefiting from the prime and others displaying negative priming, as indexed by T2/T1 in prime present – prime absent Lag 4 trials. Specifically, priming effects ranged between −21.1 and +26.1 (mean (stdev) priming magnitude: 1.5 (11.6)). We therefore first examined whether we could replicate the cross-subject relationship between distractor suppression ability and AB performance reported by Dux and Marois [1]. To this end, individual subjects' Lag4 distractor priming magnitude was correlated with individual AB recovery (Lag10-Lag4 T2/T1) in prime absent trials. Replicating Dux and Marois, we found that those individuals who displayed greater positive priming generally showed a larger AB at Lag4 (r(38) = .32, p = .047; Figure 2B). As positive priming (i.e., better T2 performance after a priming distractor) is indicative of reduced distracter inhibition, a failure to inhibit distractors was thus associated with reduced *recovery* from the AB.

One could argue that a positive priming effect will necessarily be larger in those individuals with more room for improvement (i.e., those with a large AB). To counter this possibility, we correlated distracter suppression ability with AB recovery (T2/T1 Lag10 – Lag4), while controlling for AB magnitude (T2/T1 Lag10 – Lag2). The relationship between distracter suppression ability and AB recovery remained significant as shown by a partial correlation analysis (r(38) = .34, p = .035).

Next, we examined the relationship between AB *magnitude* (Lag10 – Lag2 T2/T1) in prime absent trials and Lag4 distractor priming magnitude (T2/T1 prime present – T2/T1 prime absent). No significant cross-subject correlation was observed between distractor inhibition and AB magnitude (r(38) = .02, p = .89; Figure 2B). Thus, together the current findings indicate that a failure to suppress distracting information may delay AB recovery, but does not affect AB depth. Notably, post-hoc Spearman correlation analyses showed that neither T1 accuracy in Lag2 prime absent trials nor average T1 accuracy in prime absent trials was associated with distractor suppression ability (r(38) = −.07; p = .67 and r(38) = −.09; p = .59), indicating that inhibition did not influence T1 selection. Thus, distractor interference may have simply delayed rather than impaired T1 encoding processes (see also [13]). Yet, it is also possible that distractors interfered with T2 processing. Previous work has, for example, shown that T2 performance can be affected by target-distractor similarity, with highly similar distractors reducing T2 accuracy at longer lags [33]. Visser et al. argued that highly similar distractors may more often be processed as a target, resulting in an additional AB, which would specifically be reflected in reduced AB recovery. As in the current study, distractor inhibition was only associated with T2 accaracy at Lag4, not Lag2, it is hence possible that distractors selectively affected T2 processing at longer lags. It should be noted, however, that AB magnitude and AB recovery were modestly correlated with each other (r(39) = .38, p = .017), indicating that the two indices reflect at least in part different mechanisms. This could also explain why only AB recovery correlated with distractor suppression ability. Importantly, split-half analyses revealed that our measures of AB magnitude, AB recovery and distracter priming were reliable, as no differences between the first and second half of the task were found for any of these performance indices (all p′s >.24).

To summarize, the current findings indicate that recovery from the AB is determined, at least in part, by distractor suppression ability, for increased distractor inhibition was associated with attenuated AB recovery. A failure to inhibit distracting stimuli may slow down T1 consolidation processes through interference thereby keeping the system from proceeding to the next target task (e.g., [5], [34]) and/or could impair T2 encoding processes (e.g., [33]). Yet, our findings could be consistent with theoretical proposals that attribute the AB to the shielding of T1 processing as well [6], [35]–[37]. By preventing gating of distractor stimuli, undisturbed consolidation of T1 in working memory is promoted. In general, they are in line with previous research indicating that good filtering efficiency may benefit the AB (e.g., [1], [10]–[12], [14], [15], [21], [36], [38]).

The second aim of the current study was to replicate a previously reported association between sEBR, a marker of striatal dopaminergic functioning [25], and AB magnitude [22], and extend this finding by also examining the relationship between sEBR and AB recovery. One subject had to be excluded from the analyses concerning sEBR due to very noisy sEBR recordings, which prevented clear identification of eye blinks. In line with previous reports (e.g., [22], [28]–[30], [39]), average sEBR was 15.2 blinks per minute, with individual sEBR values ranging between 5 and 35. In contrast to Colzato et al. [22], sEBR did not predict AB size in the current study, as a cross-subject correlation analyses did not reveal a significant relationship between individual sEBR and AB magnitude (r(37) = −.13; p = .42). In addition, no association was observed between individual sEBR and AB recovery (r(37) = −.21; p = .20). One possible explanation for replication failure are differences between the specific AB tasks used in this study and the Colzato et al. study, such as differences in stimulus presentation rate (10.9 Hz (this study) vs. 12.5 Hz (Colzato et al. study)), stimulus duration (92 ms vs. 40 ms), and/or the presence of a blank between stimuli (no blank vs. 40-ms blank) – all factors that can, notably, influence target selection processes and could thereby contribute to the observed discrepancy in findings. Furthermore, in the current study, T1 and T2 were letters indicated by different colors, while in the Colzato et al. study, both the T1 and T2 task involved identifying a black letter (and ignoring black numbers). It has been suggested that a set switch between T1 and T2 may introduce an additional bottleneck in the processing stream that may mask individual differences in AB size [40]–[42] (see [24], [43] for a similar argument). Future studies are necessary to determine whether the observed relationship between markers of striatal dopaminergic functioning and AB size is specific to the AB processing bottleneck or not.

## Conclusions

In general, the current findings support an important role for distractor inhibition in the AB. More specifically, they suggest that one's ability to suppress distracting information may determine how quickly one recovers from the AB, rather than one's AB size. It should be noted that our data do not imply that other mechanisms, such as T1-induced resource depletion, do not contribute to the AB, as it is clear that many factors influence the AB.
